# In silico analysis of promoter regions and regulatory elements (motifs and CpG islands) of the genes encoding for alcohol production in *Saccharomyces cerevisiaea S288C* and *Schizosaccharomyces pombe 972h-*

**DOI:** 10.1186/s43141-020-00097-9

**Published:** 2021-01-11

**Authors:** Jemal Aman Beshir, Mulugeta Kebede

**Affiliations:** 1 Department of Applied Biology, School of Applied Natural Science, Adama Science and Technology University, P.O. Box 1888, Adama, Ethiopia; 2 Ethiopian Sugar Corporation, Sugar Academy, Wonji, Ethiopia

**Keywords:** Alcohol production, CpG islands, Motifs, *Saccharomyces cerevisiaea S288C*, *Schizosaccharomyces pombe 972h-*

## Abstract

**Background:**

The crucial factor in the production of bio-fuels is the choice of potent microorganisms used in fermentation processes. Despite the evolving trend of using bacteria, yeast is still the primary choice for fermentation. Molecular characterization of many genes from baker’s yeast (*Saccharomyces cerevisiaea*), and fission yeast (*Schizosaccharomyces pombe*), have improved our understanding in gene structure and the regulation of its expression. This in silico study was done with the aim of analyzing the promoter regions, transcription start site (TSS), and CpG islands of genes encoding for alcohol production in *S. cerevisiaea S288C* and *S. pombe 972h-*.

**Results:**

The analysis revealed the highest promoter prediction scores (1.0) were obtained in five sequences (AAD4, SFA1, GRE3, YKL071W, and YPR127W) for *S. cerevisiaea S288C* TSS while the lowest (0.8) were found in three sequences (AAD6, ADH5, and BDH2). Similarly, in *S. pombe 972h-*, the highest (0.99) and lowest (0.88) prediction scores were obtained in five (Adh1, SPBC8E4.04, SPBC215.11c, SPAP32A8.02, and SPAC19G12.09) and one (erg27) sequences, respectively. Determination of common motifs revealed that *S. cerevisiaea S288C* had 100% coverage at M*Sc*1 with an *E* value of 3.7e−007 while *S. pombe 972h-* had 95.23% at M*Sp*1 with an *E* value of 2.6e+002. Furthermore, comparison of identified transcription factor proteins indicated that 88.88% of M*Sp*1 were exactly similar to M*Sc*1. It also revealed that only 21.73% in *S. cerevisiaea S288C* and 28% in *S. pombe 972h-* of the gene body regions had CpG islands. A combined phylogenetic analysis indicated that all sequences from both *S. cerevisiaea S288C* and *S. pombe 972h-* were divided into four subgroups (I, II, III, and IV). The four clades are respectively colored in blue, red, green, and violet.

**Conclusion:**

This in silico analysis of gene promoter regions and transcription factors through the actions of regulatory structure such as motifs and CpG islands of genes encoding alcohol production could be used to predict gene expression profiles in yeast species.

## Background

The scarcity and rising prices of fossil fuels, geo-political instability in countries that hold most of the proven oil reserves together with apprehension about the environmental harm created by them, have resulted in increasing efforts to search for alternative energy sources [1]. Hence, production and use of bio-fuels for transport fuel has recently attracted significant attention worldwide. Likewise, Ethiopia’s sustainable development and the national fuel security can only be realized with increased production and utilization of renewable fuels. Substituting the demand for fossil fuel by locally produced fuels such as bio-ethanol and bio-diesel is paramount importance for the country’s economic use of scarce energy resources.

Bio-ethanol has been made since ancient times by fermenting sugars, and most bio-ethanol used for fuel and alcoholic drinks, and most industrial ethanol, is made by this process (Licht 2001; as cited in [2]). Great strides in research together with the development of new yeast strains have led to demands to model a new yeast strain which can withstand and produce at higher levels of alcohol, temperatures, and pH. This requires immense knowledge of the fermentation processes to improve its efficiency which is dependent on various factors, namely, process design, molasses quality, yeast strain, contamination, nutrient availability, and raw material purity [2]. Yeast alcohol is one of the most valuable products originating from the biotechnological industry with respect to both value and amount [3]. Yeast selection for fuel ethanol production over the past two decades and most bio-ethanol-related researches in developing tropical countries have focused primarily on the isolation of local Saccharomyces yeasts and their use for industrial ethanol production [4–6].

The development of DNA transformation in yeast has made possible the rapid molecular isolation of many genes from baker’s yeast (*Saccharomyces cerevisiaea*) and fission yeast (*Schizosaccharomyces pombe*). Concomitantly, our understanding of many aspects of gene structure and regulation of gene expression in these organisms has improved. Although the two organisms are similar in that they are both spore-forming yeasts capable of strong alcoholic fermentation, *S. pombe* actually has diverged significantly from *S. cerevisiaea* [7]. In recent years, genome mining and in silico analysis of gene sequences and their products have become a key methodology to identify gene expression patterns, sequences responsible for development of new molecules, leading to the discovery of dozens of novel compounds [8, 9]. A variety of computational tools have been developed to support scientists in this field. Most of the available tools are dedicated to the in silico analysis of specific gene and gene products [8].

Gene expression varies among tissues and even different cell types but also in response to specific signals (physiological, environmental, etc.). The main mechanism of transcriptional regulation is orchestrated by proteins called transcription factors (TFs), which promote (as activators) or block (as repressors) the recruitment of the RNA polymerase II (Pol II complex) [10]. The promoter is a DNA sequence that the transcription apparatus recognizes and binds. It indicates which of the two DNA strands is to be read as the template and the direction of transcription [11]. It is a functional region containing complex regulatory elements for determining the transcription initiation of genes [9, 10, 12, 13]. DNA-binding sites or motifs refer to short DNA sequences (typically 4 to 30 base pairs long, but up to 200 bp for recombination sites) that are explicitly bound by one or more DNA-binding proteins or protein complexes [14]. It is often associated with specialized proteins known as transcription factors and is thus linked to transcriptional regulation. A structural feature that has proven useful in the detection of promoters is the so called CpG islands, i.e., regions that are rich in CpGs, which are important because of their strong link with gene regulation[15]. CpG islands are playing an important role in gene regulation through epigenetic changes [16].

Therefore, the aim of this study is to predict promoter and regulatory elements of genes encoding alcohol production in yeast species (*Saccharomyces cerevisiaea S288C* and *Schizosaccharomyces pombe 972h-*) thereby providing basic information which could support the effort of improving them for a commercial-scale bio-ethanol production.

## Methods

### Determination of transcription start sites and promoter regions for genes encoding alcohol production

Gene sequences of yeast species (*Saccharomyces cerevisiaea S288C* and *Schizosaccharomyces pombe 972h-*) *for genes encoding alcohol production* were retrieved as FASTA file from NCBI Genome Browser (https://www.ncbi.nlm.nih.gov/gene). Gene sequences starting by ATG (starting codon) were identified, and coding sequences were used in this analysis. However, for *S. pombe 972h-*, all the sequences retrieved from direct NCBI web were not having the functional gene structure (no ATG in the beginning and many stop codons in the middle). Therefore, sequences used for the current study were retrieved via NCBI Reference Sequences (RefSeq). This section includes genomic Reference Sequences (RefSeqs) from all assemblies on which these genes were annotated, such as RefSeqs for chromosomes and scaffolds (contigs) from both reference and alternate assemblies. To this end, gene sequences were taken as identical protein annotated from PomBase annotation Provider for Eukaryotic Annotation Propagation Pipeline.

Generally, 23 and 21 sequences of alcohol dehydrogenase were retrieved for *S. cerevisiaea S288C* and *S. pombe 972h-*, respectively. To determine respective transcriptional start sites (TSSs) for all gene sequences, about 1-kb sequences upstream of the start codon were excised from all genes except for *ADH1* of *S. cerevisiaea S288C* which was at 2-kb upstream of start codon. Similarly, all gene sequences of *S. pombe 972h-*, except three genes (erg27 at 2.5 kb and SPBC8E4.04 and SPBC337.11 at 2 kb), had TSSs at 1-kb sequences upstream of their start codons.

The Neural Network Promoter Prediction (NNPP version 2.2) tool set was used with the minimum standard predictive score (between 0 and 1) [17]. For those regions containing more than one TSS, the one with the highest value of prediction score was considered to have trustable and truthful prediction. Promoter regions were defined as 1-kb region upstream of each TSS. For those regions containing more than one TSS, the highest value of prediction score will be considered so as to have a more accurate prediction.

### Determination of common motifs and TFs for genes encoding alcohol production in the promoter region

Analysis of conserved motifs for genes encoding for alcohol productions for both yeast species was performed by MEME (Multiple Em for Motif Elicitation) software version 3.5.4 (http://meme.sdsc.edu). This online web-based analysis was performed with minimum and maximum motif width of 6 and 50 residues, respectively, for both yeast species whereas a maximum number of motifs for *S. cerevisiaea S288C* and *S. pombe 972h-* were 23 and 21, respectively, which were used to identify probable promoter regulatory elements (motifs), keeping the rest of the parameters at default. The MEME output in HTML showed the motifs as local multiple alignments of the input sequences, as well as in several other formats. Buttons on the MEME HTML output were allowed one or all of the motifs to be forwarded for additional investigation. Descriptions the identification of motifs by TOMTOM [18] web server were designated where numerous sequence databases can be searched for sequences matching the identified motif, in which the output of TOMTOM will include LOGOS on behalf of the alignment of two motifs, the *p* value and *q* value (a measure of false discovery rate) of the match [18]. TOMTOM showed that the query motif closely resembles the binding motif in the set of genes encoding for alcohol production promoter regions.

### Search for CpG islands for genes encoding alcohol production promoter regions

To search CpG islands, first, the stringent search criteria were used in the Takai and Jones algorithm: GC content ≥ 50%, Obs CpG/ExpCpG ≥ 0.60, and length ≥ 200 bp [20]. For this purpose, the CpG island searcher program (CpGi130) available at web link (http://www.bioinformatics.org/sms/cpg_island.html) was used. The CpG island graphs were plotted using EMBOSS Cpgplot (https://www.ebi.ac.uk/Tools/seqstats/emboss_cpgplot/) which identify and plot CpG islands in nucleotide sequence(s). Secondly, the CLC Genomics Workbench ver. 3.6.5 (http://clcbio.com, CLC bio, Aarhus, Denmark) was used for restriction enzyme M*Sp*I cutting sites (fragment sizes between 40 and 220 bp).

### Phylogenetic analysis

Phylogenetic analysis of gene sequences of both yeast species (*S. cerevisiaea S288C* and *S. pombe 972h-*) was conducted using the Molecular Evolution Genetic Analysis 6 (MEGA6) tool by the neighbor-joining tree-making method. Similarly, Tajima’s neutrality test of selection was conducted using the same software to find nucleotide diversity. The p-distance model was applied with transition and trans-version nucleotide substitution. Bootstrap values of the super tree were computed with 2000 repetitions with uniform rate among sites and complete deletion of gaps/missing data were used to analyze the sequences.

## Results

### Determination of transcription start sites and promoter regions for genes encoding alcohol production

Promoter region analysis of genes encoding for alcohol productions of both yeast species (*S. cerevisiaea S288C* and *S. pombe 972h-*) showed a great variation in the number of TSS. The highest promoter prediction scores (1.0) for TSS of *S. cerevisiaea S288C* alcohol dehydrogenase were obtained for five gene sequences (AAD4, SFA1, GRE3, YKL071W, andYPR127W) while the lowest promoter prediction scores (0.8) were obtained for three gene sequences (AAD6, ADH5, and BDH2) (Table 1). In addition, the result of promoter predictions for *S. cerevisiaea S288C* sequences with score cutoff 0.80 showed that out of twenty-three gene sequences used in this analysis only ADH1 and ADH7 (8.70%) had showed a single TSS while the remaining (91.30%) showed multiple TSS. In these scenarios, TSSs with the highest prediction scores were considered for further uses. TSSs of genes encoding for alcohol production in *S. cerevisiaea S288C* were mostly located in the upstream region of − 31 to − 1545 bp, with the relatively highest incidence of occurrence in the upstream region of − 1 to − 200 bp (10 sequences, *43%*) followed by − 201 to − 400 bp and − 601 to − 800 bp (4 sequences each, *17.4%*) from the transcription start site, while the lowest occurrence was observed at − 801 to − 1000 bp and above − 1000 bp (only 1 sequence each).

**Table 1:** TSS number, its promoter predictive score values, and distance from the start codon of *S. cerevisiaea S288C*

Gene name	Gene ID	Location	Number of TSS	Predictive score at cutoff value of 0.8	Distance from start codon (ATG)
Alcohol dehydrogenase ADH1	854068	Chromosome XV; NC_001147.6 (c160594-159548)	1	*0.99*	− *1545*
Alcohol dehydrogenase ADH2	855349	Chromosome XIII; NC_001145.3 (c874337-873291)	4	*0.98*, 0.93, 0.92, 0.91	− *130*, − 467, − 197, − 177
S-(Hydroxymethyl) glutathione dehydrogenase SFA1	851386	Chromosome IV; NC_001136.10 (159604-160764)	3	*1.00*, 0.94, 0.89	− *789*, − 159, − 531
Alcohol dehydrogenase ADH3	855107	Chromosome XIII; NC_001145.3 (434788-435915)	3	*0.91*, 0.89, 0.88	− *31*, − 639, − 657
Alcohol dehydrogenase ADH4	852636	Chromosome VII; NC_001139.9 (15159-16307)	2	*0.89*, 0.86	− *95*, − 120
Alcohol dehydrogenase ADH5	852442	Chromosome II, NC_001134.8 (533762-534817)	6	*0.96*, 0.91, 0.86, 0.84, 0.8, 0.8	− *90*, − 737, − 224, − 703, − 688, − 715
Alcohol dehydrogenase ADH6	855368	Chromosome XIII, NC_001145.3 (c912143-911061)	3	*0.94*, 0.93, 0.89	− *215*, − 822, − 71
NADP-dependent alcohol dehydrogenase (ADH7)	850469	Chromosome III, NC_001135.5 (309070-310155)	1	*0.93*	− *63*
Putative aryl-alcohol dehydrogenase (AAD3)	850471	Chromosome III, NC_001135.5 (313890-314981)	2	*0.98*, 0.98	− *133*, − 199
Putative aryl-alcohol dehydrogenase AAD4	851354	Chromosome IV, NC_001136.10 (c17577-18566)	4	*1.00*, 0.96, 0.92, 0.90	− *759*, − 356, − 245, − 42
Aryl-alcohol dehydrogenase (NADP+) activity (AAD6)	850488	Chromosome VI, NC_001138.5 (c14305-15431)	5	*0.97*, 0.89, 0.86, 0.85, 0.80	− *101*, − 518, − 734, − 457, − 28
Putative aryl-alcohol dehydrogenase AAD10	853620	Chromosome X, NC_001142.9 (727405-728271)	3	*0.97*, 0.86, 0.83	− *654*, − 359, − 171
Putative aryl-alcohol dehydrogenase AAD14	855385	Chromosome XIV, NC_001146.8 (c16118-17248)	2	*0.94*, 0.81	− *663*, − 452
Putative aryl-alcohol dehydrogenase AAD15	853999	Chromosome XV, NC_001147.6 (c1647-2078)	2	*0.94*, 0.86	− *492*, − 955
D-Arabinose 1-dehydrogenase (NAD(P)(+)) ARA1	852446	Chromosome II, NC_001134.8 (539987..541021)	2	*0.98*, 0.95	− *147*, − 92
Glucose-6-phosphate dehydrogenase (ZWF1)	855480	Chromosome XIV, NC_001146.8 (c196426..197943)	4	*0.99*, 0.94, 0.86, 0.85	− *318*, − 145, − 636, − 120
Glycerol 2-dehydrogenase (NADP(+)) GCY1	854287	Chromosome XV, NC_001147.6 (551114-552052)	4	*0.99*, 0.97, 0.92, 0.91	− *357*, − 85,− 899, − 829
Trifunctional aldehyde reductase/xylose reductase/glucose 1-dehydrogenase (NADP(+))GRE3	856504	Chromosome VIII, NC_001140.6 (323409-324392)	3	*1.00*, 0.96, 0.90	− *355*, − 778, − 679
Putative dehydrogenase BDH2	851238	Chromosome I, NC_001133.9 (33448-34701)	2	*0.99*, 0.8	− *146*, − 154
Glucose 1-dehydrogenase (NADP(+)) YPR1	851974	Chromosome IV, NC_001136.10 (1213904-1214842)	3	*0.87*, 0.83, 0.83	− *97*, − 10, − 252
Hypothetical protein YKL071W	853792	Chromosome XI, NC_001143.9 (305114-305884)	3	*1.00*, 0.95, 0.93	− *73*, − 513, − 881
Hypothetical protein YFL041W-A	1466401	Chromosome VI, NC_001138.5 (48734..48925)	2	*0.97*, 0.84	− *866*, − 115
Pyridoxine 4-dehydrogenase (YPR127W)	856245	Chromosome XVI, NC_001148.4 (790083..791120)	2	*1.00*, 0.83	− *437*, − 776

*Note*: Figures in italics were taken as cutoff point and respective distances for more than one TSS

Likewise, twenty-one sequences of genes encoding for alcohol production for *S. pombe 972h-* (fission yeast) were retrieved from NCBI Genome Browser. Accordingly, the result of TSS and promoter analysis had showed a significant variation in the number of TSS and the distance of TSS from the start codons (Table 2). The numbers of TSSs were varied from 1 to 3 with majority of sequences (*71.43%*) having more than one TSS. In particular to this fact, out of twenty-one sequences six, seven, and eight sequences had one, two, and three TSSs respectively. The relative locations of all TSS with respect to start codon were given in Table 2. The nearest TSS were recorded for SPCC13B11.04c (− 39) followed by Adh1 (− 77) while the far-flanged TSS were observed for egr27 (− 2308) followed by SPBC337.11 (− 1636) upstream of the start codons of their respective genes. The current analysis also revealed that the relatively highest frequency of occurrence in the upstream region of − 1 to − 200 bp and − 201 to − 400 bp (6 sequences each, 57.1% share) followed by − 401 to − 600 bp (4 sequences, 19.04%) from the transcription start site, while the lowest occurrences were observed at − 601 to − 800 and − 801 to − 1000 bp (only 1 sequence each) and whereas three sequences had their TSS at above − 1000 bp (Table 1). The predictive score at a cutoff value of 0.8 ranged from 0.99 to 0.88.

**Table 2:** TSS number, its promoter predictive score values, and distance from start codon for *S. pombe 972h-*

Gene name	Gene ID	Location	Number of TSS	Predictive score at cutoff value of 0.8	Distance from start codon (ATG)
Alcohol dehydrogenase Adh1	2538902	Chromosome III, NC_003421.2 (158942-1592889)	1	*0.99*	− 77
Alcohol dehydrogenase Adh4	2542714	Chromosome I, NC_003424.3 (c156430-158279)	2	*0.98*, 0.89	− 565, − 85
Putative alcohol dehydrogenase (SPBC1773.06c)	2540139	Chromosome II, NC_003423.3 (c293225-294725	2	*0.97*, 0.87	− 120, − 39
Pyridoxal reductase Plr1	2542917	Chromosome I, NC_003424.3 (4461697-4462901)	2	*0.95*, 0.90	− 89, 246
Putative aldo/keto reductase (SPBC8E4.04)	2541256	Chromosome II, NC_003423.3 (c4432247-4433501)	1	*0.99*	− 1632
Putative glutathione-dependent formaldehyde dehydrogenase SPCC13B11.04c	2538802	Chromosome III, NC_003421.2 (c1596549-1599028)	3	*0.92*, 0.80, 0.80	− 39, − 59, − 295
NADH/NADPH-dependent indole-3-acetaldehyde reductase AKR3C2 (SPAC19G12.09)	2542483	Chromosome I, NC_003424.3 (4059980-4061192)	3	*0.99*, 0.96, 0.92	− 211, − 42, − 560
Putative dehydrogenase SPAC2E1P3.01:	2541784	Chromosome: I; NC_003424.3 (2922761..2924720)	2	*0.98*, 0.95	− 126, − 848
Putative 3-oxoacyl-[acyl-carrier-protein] reductase Oar2	2543512	Chromosome: I; NC_003424.3 (c3180426-3181164)	3	*0.97*, 0.89, 0.83	− 402, − 746, 795
Aldose reductase ARK13 family YakC (yak3)	2541648	Chromosome I, NC_003424.3 (4248777-4250231)	1	0.92	− 260
Putative xylose and arabinose reductase (SPAP32A8.02)	2541584	Chromosome I, NC_003424.3 (3405955-3409780)	2	*0.99*, 0.97	− 273, − 353
NADP-dependent glucose 1-dehydrogenase (SPAC26F1.07)	2542088	Chromosome I; NC_003424.3 (c5170789..5172112)	3	*0.98*, 0.96, 0.84	− 227, − 47, 290
Hypothetical protein SPBC16A3.02c	2540039	Chromosome II, NC_003423.3 (4296979-4298874)	3	*0.97*, 0.93, 0.86	− 493, − 602, − 731
Aldo-keto reductase family protein (SPAC977.14c)	2543325	Chromosome I, NC_003424.3 (c59614-60907)	1	*0.89*	− 783
Putative pyridoxal reductase (SPCC1281.04)	2539165	Chromosome III, NC_003421.2 (1386524-1387961)	3	*0.95*, 0.90, 0.87	− 291, − 345, − 433
Aldo/keto reductase family protein (SPAC750.01)	3361570	Chromosome I, NC_003424.3 (5555716-5556768)	1	*0.98*	− 927
Putative xylose and arabinose reductase (SPAC2F3.05c)	2541958	Chromosome I, NC_003424.3 (c3928625-3929950)	2	*0.92*, 0.81	− 520, − 494
Putative pyridoxal reductase (SPAC3A11.11c)	2543167	Chromosome I, NC_003424.3 (3446262-3448081)	2	0.97, 0.95	− 146, − 878
Aldo/keto reductase family protein (SPBC215.11c)	2540698	Chromosome II, NC_003423.3 (c4050360-4051532)	3	*0.99*, 0.97, 0.94	− 380, − 688, − 753

*Note*: Figures in italics were taken as the cutoff point and respective distances for more than one TSS

### Determination of common motifs and TFs for genes encoding alcohol production in the promoter region

The promoter regions shared by majority of the gene sequences used in the current study revealed that *S. cerevisiaea S288C* had 100% coverage among the gene sequences at M*Sc*1 with an *E* value of 3.7e−007 and 15 motif widths (Table 3).

**Table 3:** List of discovered motifs and the number and proportion of promoter-containing motifs for *S. cerevisiaea S288C*

Discovered motif	Number (%) of promoter-containing motifs	*E* value	Motif width	Total number of binding sites
M*Sc*1	23 (100%)	3.7e−007	15	23
M*Sc*2	18 (78.26%)	1.8e+004	29	18
M*Sc*3	8 (34.78%)	2.5e+004	10	8
M*Sc*4	3 (13.04%)	4.8e+004	29	3
M*Sc*5	7 (30.43%)	1.6e+004	29	7

M*Sc* motif for *S. cerevisiaea S288C*

**Fig. 1:**
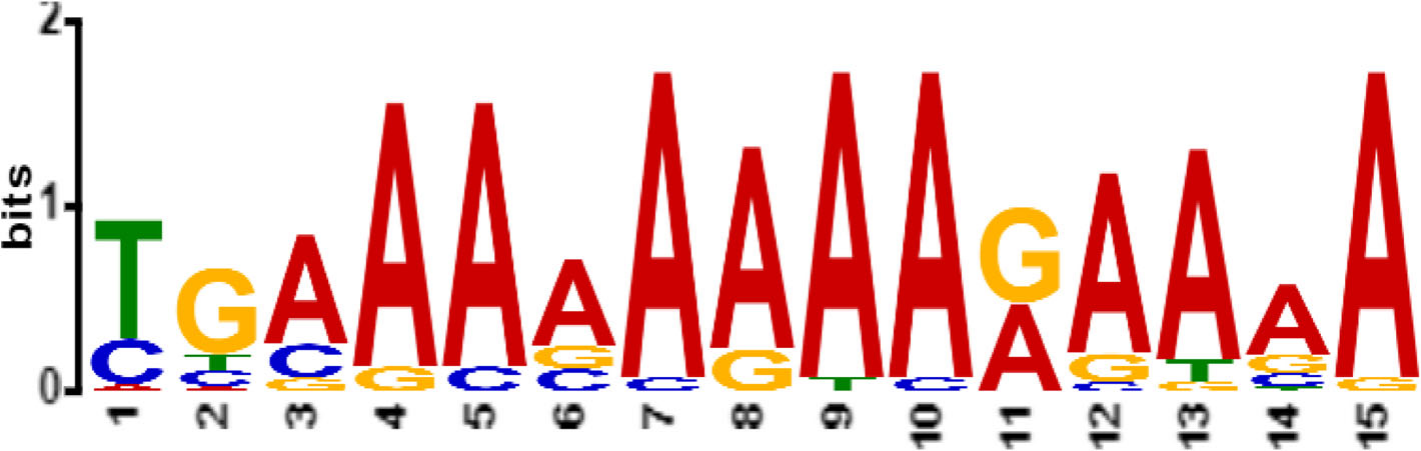
Sequence logo for the identified common promoter motif M*Sc*1 gene for gene encoding alcohol production gene in *S. cerevisiaea S288C*

To determine motifs which are functionally important, motifs which were shared by majority of promoter regions of *S. cerevisiaea S288C* genes encoding for alcohol production were chosen. Accordingly, M*Sc*1 was revealed as the common promoter motif for all (100%) genes that serves as binding sites for transcription factors involved in the expression regulation of these genes. Sequence logo for M*Sc*1 generated by MEME is presented in Fig. 1

**Fig. 2:**
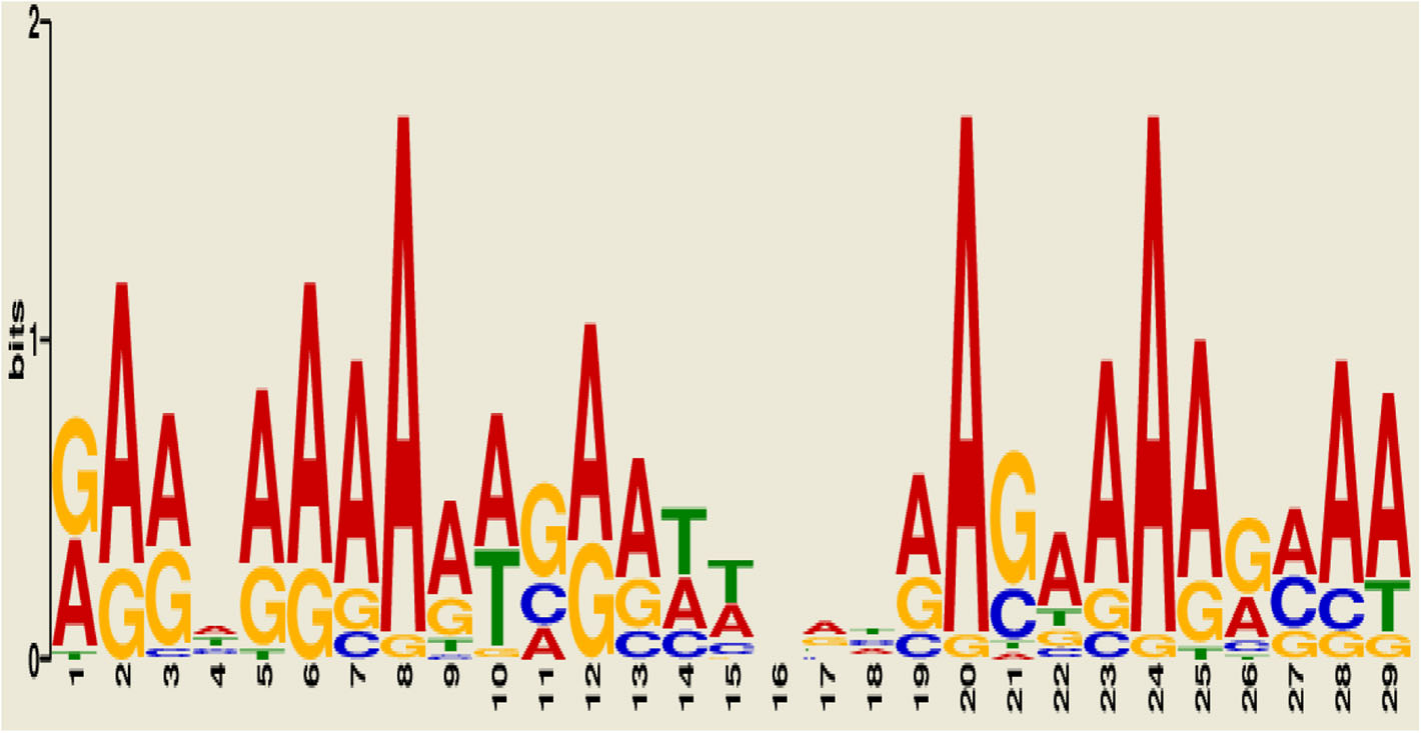
Sequence logo for the identified common promoter motif M*Sp*1 for gene encoding alcohol production gene in *S. pombe*

Likewise, *S. pombe 972h-* promoter sequences had 95.23% conserved motif at M*Sp*1 with *E* value of 2.6e+002 and 29 motif widths (Table 4). The common motifs of M*Sp*1 shared by majority of the genes encoding for alcohol production sequences of *S. pombe 972h-* as generated by MEME revealed in Fig. 2.

**Table 4:** List of discovered motifs and the number and proportion of promoter-containing motifs for *S. pombe 972h-*

Discovered motif	Number (%) of promoter-containing motifs	*E* value	Motif width	Total number of binding sites
*MSp*1	20 (95.23%)	2.6e+002	29	20
M*Sp*2	4 (19.04%)	1.5e+004	48	4
M*Sp*3	13 (61.90%)	1.8e+004	15	13
M*Sp*4	6 (28.57%)	2.2e+004	21	6
M*Sp*5	5 (23.81%)	1.8e+005	19	5

M*Sp* motif for *S. pombe*

Furthermore, candidate transcription factor proteins were identified for motifs (M*Sc*1 and M*Sp*1) of both yeast species. They were then compared to the registered motifs in publicly available database of Jaspar2018_Core_Fungi_Non-Redundant DNA so as to see if they are similar to known regulatory motifs using TOMTOM web application [19]. The output from TOMTOM also links back to the parent motif database for more detailed information on biological functions of the matched motif. As a result, 13 motifs out of 176 common promoter motif/transcription factors were identified for M*Sc*1 while only 9 motifs out of 176 in M*Sp*1 were being found matched with known motifs found in JASPAR 2018 CORE fungi motif databases (Table 2). In addition, the comparison of the identified transcription factor proteins indicated that almost 88.88% (8 out of 9) of the M*Sp*1 were exactly similar to that of M*Sc*1. This could be due to the fact that these two yeast species (*S. cerevisiaea S288C* and *S. pombe 972h-*) shared common promoters and their subsequent transcription factor protein were highly conserved regions of the genes encoding for alcohol production. TOMTOM is a motif comparison algorithm that ranks the target motifs in a given database according to the estimated statistical significance of the match between the query and the target. In similar manner, TOMTOM provides LOGOS that represents the alignment of two motifs and a numeric score for the match between two motifs together with a statistical significance [21].

**Fig. 3:**
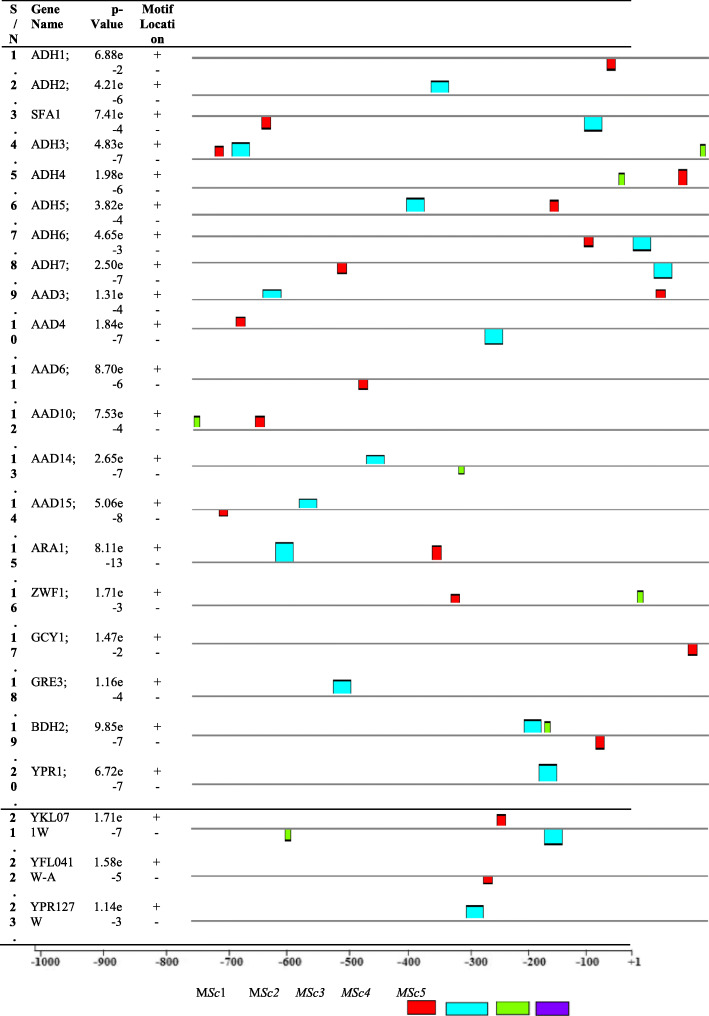
The relative positions of motifs in alcohol dehydrogenase for *S. cerevisiaea S288C* sequences relative to TSSs

**Fig. 4:**
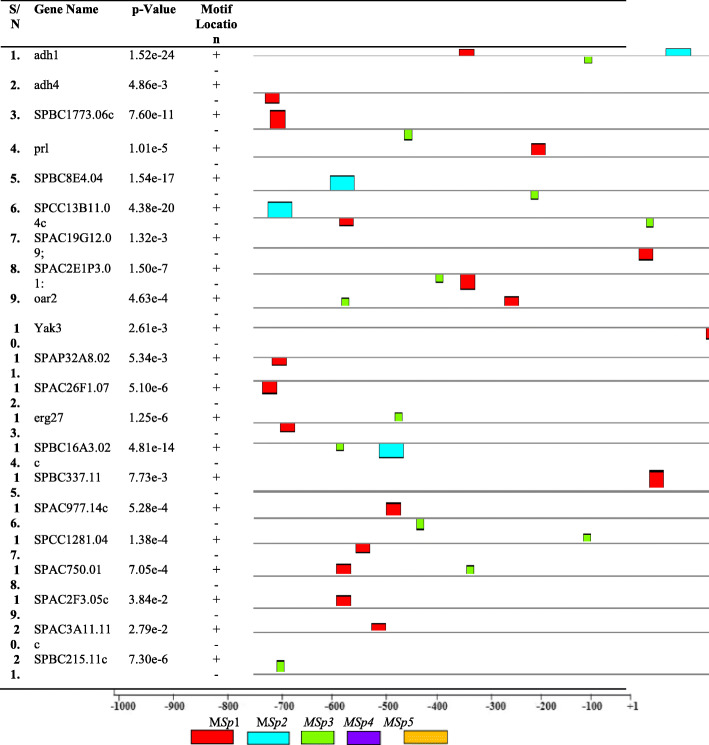
The positions and strand orientation of motifs of *S. pombe* alcohol production gene relative to TSSs

The total numbers of motifs discovered in *S. cerevisiaea S288C* for genes encoding alcohol production promoter regions were about 60 out of which relatively, higher distributions of motifs were found also in positive (39) than in negative (21) strands (Fig. 3). The location and distribution of these motifs were ranged from − 998 to − 1 while higher concentration of motifs was found between − 850 and − 50 bp of the transcription start sites (TSSs). In the same view, only 48 motifs were discovered in *S. pombe 972h-* out of which relatively, higher distributions of motifs were found also in negative (25) than in positive (23) strands (Fig. 4). The location and distribution of these motifs were ranged from − 996 to − 2 while higher concentration of motifs was found between − 800 and − 100 bp of the transcription start sites (TSSs).

### Search for CpG islands for genes encoding alcohol production in the promoter regions

**Fig. 5:**
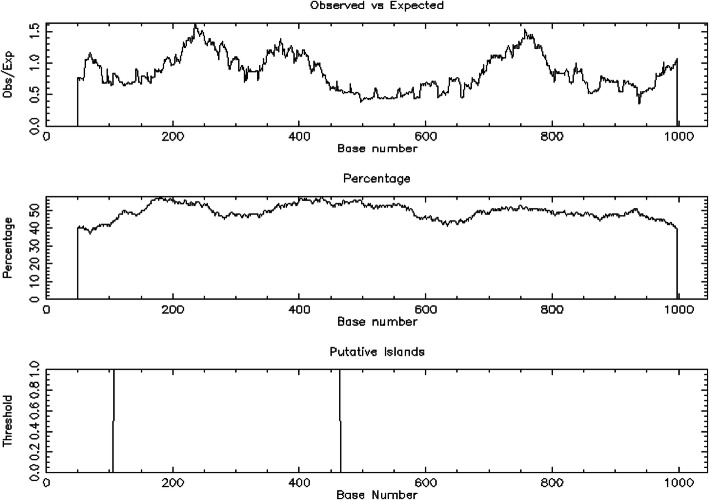
CPGPLOT islands of ADH1, with length 358 (108–465)

In the current study, revealed CpG islands were examined in the promoter and gene body regions of both yeast species using both Takai and Jones algorithm using parameters as indicated in this section of this study and CLC Genomics Workbench ver. 3.6.5. Accordingly, as per the stringent criteria of Takai and Jones [17] as indicated in this section, there were only five (ADH1 (Fig. 5), ADH2, ADH5, ZWF1, and BDH2) (21.73%) CpG islands observed in the gene body regions in analogous to only six (ADH1, SFA1, ADH3, ZWF1, BDH2, and YPR127W) out of twenty-three (26.08%) gene sequences used for the analysis in promoter regions of *S. Cerevisiaea S288C* yeast species. Likewise, only one (adh1) had CpG island in the promoter region and six (adh1, SPBC1773, SPCC13B11.04c, SPAC2E1P3.01, Yak3, and SPBC16A3.02c) CpG islands were observed in the gene body of genes encoding for alcohol production of *S. pombe 972h-*.

**Fig. 6:**
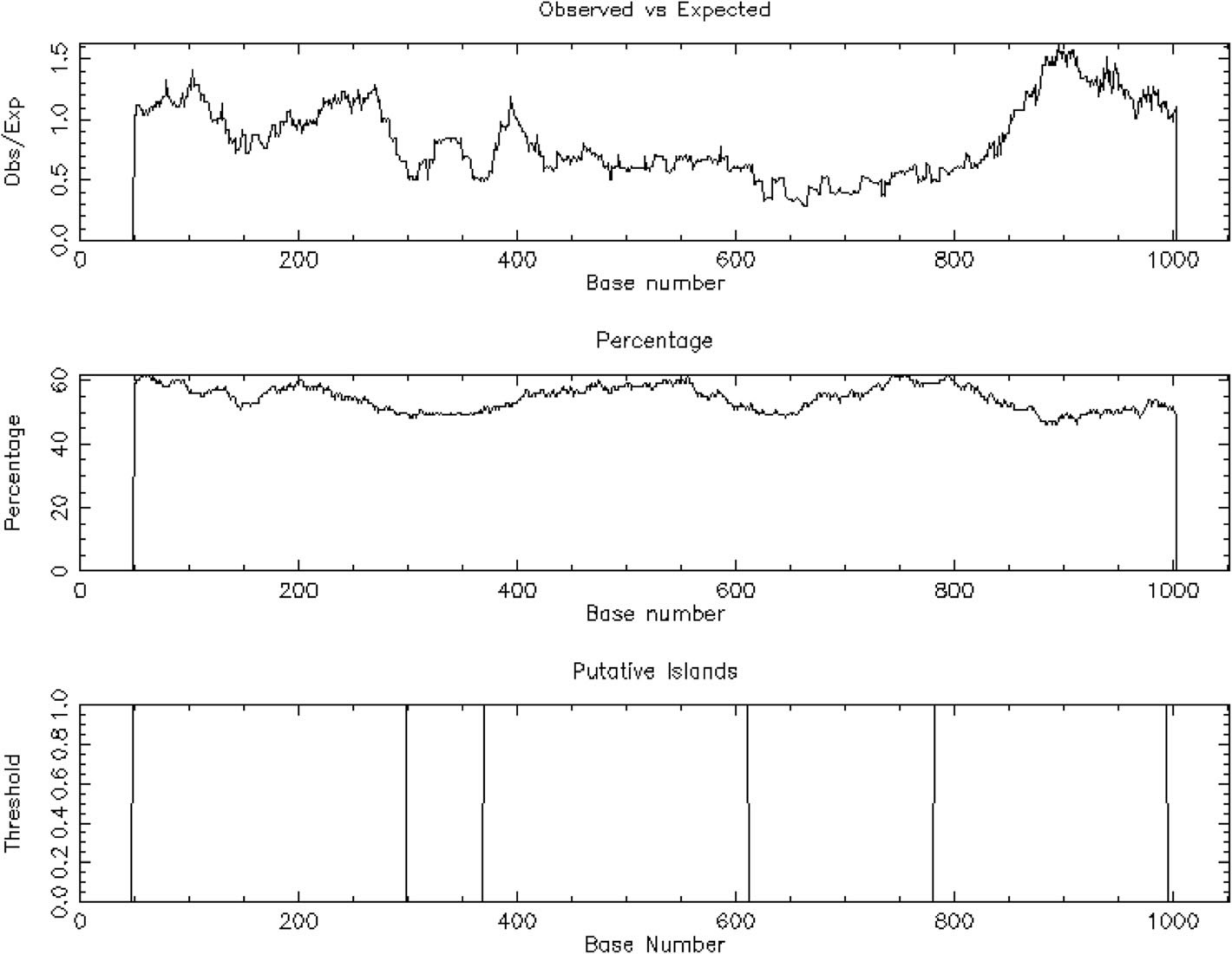
CPGPLOT islands of three regions of adh1 gene; from 1 to 1053 with length 251 (49–299), length 243

On the other hand, CLC genomics workbench ver 3.6.1 using restriction enzyme M*Sp*I (C/CGG sequence) cutting sites with standard fragment sizes between 40 and 220 bp revealed that in *S. cerevisiaea S288C* five (*ARA1*, *BDH2*, *GCY1*, *SFA1*, and *GRE3*) and only one (*GRE3*) CpG islands were found in gene body and promoter regions, respectively. In contrarily, the number of CpG islands in the gene body was only one (SPBC16A3.02c) while four (adh1 (Fig. 6), adh4, SPAP32A8.02, and SPBC337.11) in the promoter regions of *S. pombe 972h-* in alcohol dehydrogenase (Table 5). This indicates the poor occurrence of CpG islands in both gene body and promoter regions which may affect the access of promoter region of genes to their transcription factors, hence preventing their expression.

**Table 5:** Identification of M*Sp*I cutting sites and fragment sizes for genes encoding for alcohol production body and promoter regions of *S. cerevisiaea S288C* and *S. pombe 972h-*

*S. Cerevisiaea S288C*					*S. pombe 972h-*				
	Gene body		Promoter regions		Sequence name	Gene body		Promoter regions	
Sequence name	No. of cut sites (cut position/s)	Fragment size	No. of cut sites (cut position/s)	Fragment size		No. of cut sites (cut position/s)	Fragment size	No. of cut sites (cut position/s)	Fragment size
ADH1			1 (632)	–	adh1			2 (644, 743)	99
ADH3	0	–	1 (827)	–	adh4	0	–	3 (71, 501, 570)	69
ADH4	1 (146)	–	2 (276, 650)	–	SPBC1773.06c	1 (940)	–	0	–
ADH5	2 (88, 539)	–	1 (827)	–	prl	1 (289)	–	0	–
ADH6	1 (992)	–	2 (271, 979)	–	SPBC8E4.04	0	–	1 (736)	–
ADH7	0	–	1 (507)	–	SPCC13B11.04c	1 (553)	–	3 (95, 316, 506)	–
AAD10	0	–	1 (489)	–	SPAC19G12.09	1(401)	–	0	–
AAD14	3 (74, 314, 991)	–	1 (440)	–	SPAC2E1P3.01	1 (825)	–	0	–
AAD15	1 (322)	–	0	–	Yak3	1 (511)	–	0	–
AAD3	0	–	0	–	SPAP32A8.02	1 (353)	–	2 (886, 933)	47
AAD4	2 (176, 928)	–	2 (236, 902)	–	SPAC26F1.07	2 (74, 941)	–	0	–
AAD6	1 (1065)	–	1 (428)	–	erg27	2 (29, 787)	–	0	–
ARA1	2 (307, 454)	147	0	–	SPBC16A3.02c	2 (238, 431)	193	0	–
BDH2	3 (315, 481, 806)	166	2 (411, 918)	–	SPBC337.11	2 (331, 577)	–	2 (855, 948)	93
GCY1	2 (697, 737)	40	2 (198, 977)	–	SPAC977.14c	0	–	1 (739)	–
GRE3	1 (456)	–	2 (331, 496)	185	SPCC1281.04	0	–	0	–
SFA1	2 (221, 326)	105	1 (273)	–	SPAC750.01	0	–	0	–
YFL041W–A	0	–	1 (768)	–	SPAC2F3.05c	0	–	0	–
YKL071W	1 (550)	–	1 (142)	–	SPAC3A11.11c	1 (809)	–	1 (942)	–
YPR127W	1 (868)	–	0	–	SPBC215.11c		–	0	–
YPR1	2 (206, 268)	62	1 (81)	–					–

### Phylogenetic analysis

**Fig. 7:**
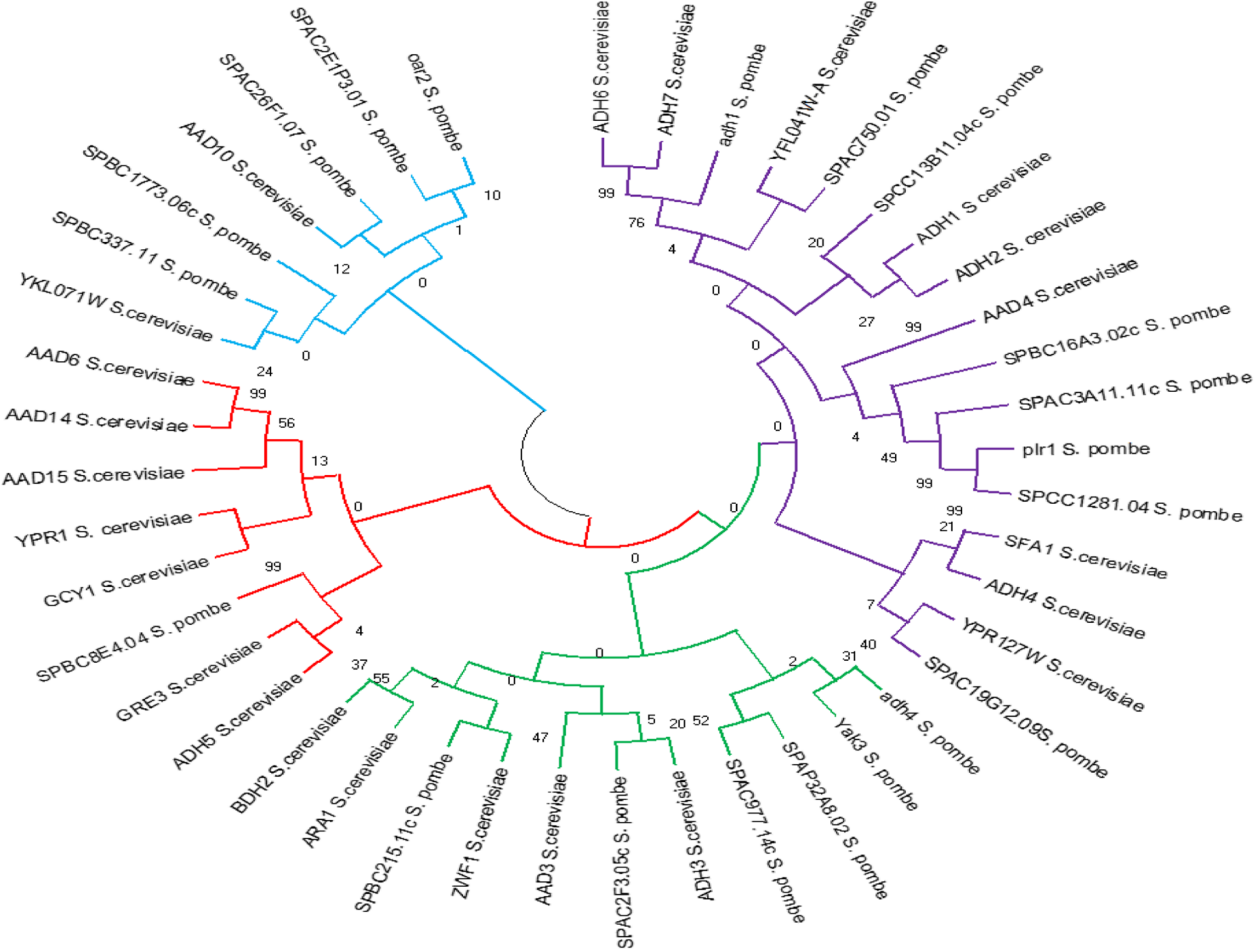
Phylogenetic tree of genes encoding alcohol production sequence from S*. cerevisiaea S288C* and *S. pombe 972h-*

A combined analysis of all the data weighted equally resulted in a single most-parsimonious cladogram (Fig. 7). Statistics for this tree revealed that in general, relationships on the tree are very strongly supported. A phylogenetic tree was generated using the neighbor-joining (NJ) as well as minimum-evolution method of MEGA 6.0. As illustrated in Fig. 7, all sequences from both *S. cerevisiaea S288C* and *S. pombe 972h*- were divided into four subgroups (I, II, III, and IV). The four clades are respectively colored in blue, red, green, and violet. The phylogenetic tree indicated that some gene sequences irrespective of source organism clustered together within each sub-group suggest a close evolutionary relationship among the genes rather than the whole species.

## Discussion

Sequence-specific DNA-binding transcription factors (TFs) are often termed as “master regulators” which bind to DNA and either activate or repress gene transcription. Determination of a gene’s transcriptional start site underlies the identification of the proximal promoter region and thus facilitates the subsequent analysis of gene expression. In the current in silico analysis, majority of the sequences in both species had multiple transcription start sites. This could give the alternative transcription potential for the gene sequence under consideration. However, for better prediction, TSS with a higher cutoff value was considered in the current study. Many authors [22–24] have reported the presence of multiple transcription sites in genes encoding for alcohol production. The comparison of the two yeast species with this regard showed that *S. cerevisiaea S288C* had a nearby site than *S. pombe 972h-.* This may be due to the fact that *S. cerevisiaea S288C* heavily relies on genes encoding for alcohol production to convert aldehydes and ketones into alcohols and NADH to NAD^+^ that the yeasts can use for energy [25]. This process of yeasts turning aldehydes and ketones into alcohols is called fermentation. In general, promoter regions are located at the immediately upstream of a transcription start site (TSS) and have a variety of sequence motifs that participate in gene regulation [26].

The establishment and maintenance of temporal and spatial patterns of gene expression are achieved primarily by transcription regulation. Functionally important motifs are usually short conserved sequence pattern associated with distinct functions of DNA often serve as transcription factor biding sites. In the current in silico analysis using MEME, the conserved short sequences of S. cerevisiaea S288C had confirmed to have higher occurrencesthan that of S. pombe 972h-. Common motifs, short DNA segments, are binding sites for transcription factors [27]. In both cases, the probability of finding a well-conserved pattern in random sequences as evidenced by an *E* value is significantly higher than expected value. It is generally believed that genes having similar expression patterns contain common motifs in their promoter regions [28, 29]. Common promoter motifs are the key signatures for a family of co-regulated genes and are usually present in the regions where complex protein interactions occur [30]. However, in some cases, single motifs can bind various transcription factors thereby bringing the genes under multiple regulatory controls [31, 32]. Extensive studies on 500 bp upstream regions of yeast promoters suggest that regulatory elements are commonly present in those regions [33].

It is well reported that CGIs are highly involved in gene regulatory processes [34]. They are present at or near the gene’s transcription start site and are often associated with the promoters of most house-keeping genes and many tissue-specific genes, and thus have important regulatory functions and can be used as gene markers [35].

A phylogenetic tree generated in the current study showed that all sequences from both *S. cerevisiaea S288C* and *S. pombe 972h-* were divided into four subgroups. This could be due to the fact that some vital genes for alcohol fermentation of yeast strains. Similar studies by other authors [34, 35] have revealed that one organism which is equally amenable to genetic manipulation as is *S. cerevisiaea* is the fission yeast *S. pombe*. Although the two organisms are similar in that they are ascospore-forming yeasts capable of strong alcoholic fermentation, *S. pombe* actually has diverged significantly from *S. cerevisiaea* [36, 37]. In fact, the available information suggests that *S. pombe* may actually be more closely related to filamentous fungi such as Neurospora and Asperglus than it is to budding yeast [9, 38].

## Conclusion

The enhancement of DNA transformation in yeast has resulted in revolutionized molecular tools and facilitated ease of thought of aspects of gene structure and regulation of gene expression of many genes from baker’s yeast (*S. cerevisiaea*) and fission yeast (*S. pombe*). Generally, the regulation of alcohol dehydrogenase enzyme which is critically important for the survival and enhanced efficiency of yeast species can better be examined and explained with the use of increasing technological advancement, genome mining, and in silico analysis of gene sequences. Majority of the genes encoding for alcohol production sequences have multiple TSS in both yeast species suggesting alternative gene regulation.

The result of this analysis could be critically important to understand the nature of promoter regions, the motif discovered in line with the transcription factor binding proteins, and the strength of the genome to different transcriptions via following the frequency of CpG islands. The phylogenetic analysis revealed that majority of genes is clustered together irrespective of the source organism suggesting a close evolutionary relationship among the gene rather than the whole species.

In general, this in silico analysis of genes encoding for alcohol production of *S. cerevisiaea S288C* and *S. pombe* could be helpful to add knowledge about the species molecular data and supportive to identify gene regulatory elements in the promoter regions. It could also help to predict gene expression profiles in various yeast species which in turn could be helpful to improve efficiencies of these organisms for domestic and commercial production of bio-fuel with higher rate of recovery.

## Data Availability

The qualitative and quantitative data of this manuscript are available through the first author.

## Abbreviations

TSS: Transcription start site M*Sc*1 Motif of *Saccharomyces cerevisiaea* 1 M*Sp*1 Motif of *Schizosaccharomyces pombe* *S. cerevisiaea* *Saccharomyces cerevisiaea* *S. pombe* *Schizosaccharomyces pombe* TFs Transcription factors MEME Multiple Em for Motif Elicitation NCBI National Center for Biotechnology Information bp Base pair NNPP Neural Network Promoter Prediction
